# Supplementary material for: Engineering a Dual Specificity γδ T-Cell Receptor for Cancer Immunotherapy
Source: Biology (Basel). 2024 Mar 20;13(3):196. doi: 10.3390/biology13030196 (PMC10968115; doi:10.3390/biology13030196)
Supplement: Supplementary file 1 [file biology-13-00196-s001.zip › biology-2891729-supplementary.pdf]

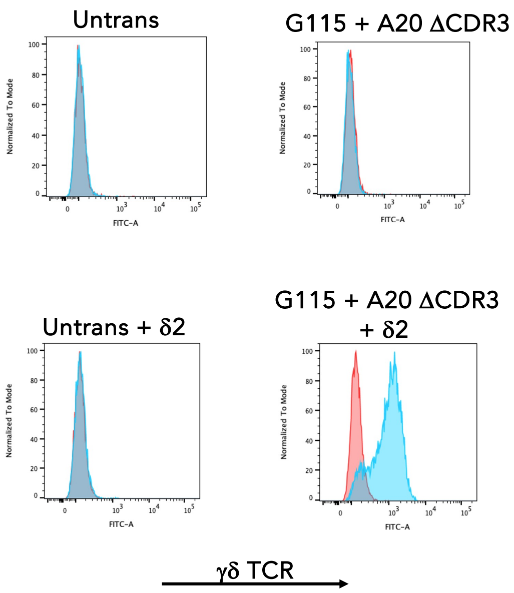

Supplementary Figure S1. Evaluation of G115 + A20 ΔCDR3 TCR expression in Jurkat cells. Representative flow cytometry plots that demonstrate cell surface γδ TCR expression in Jurkat cells following transduction with G115 + A20 ΔCDR3 alone or followed by an unmodified G115 δ2 chain. Data are representative of 3 independent replicates.

A

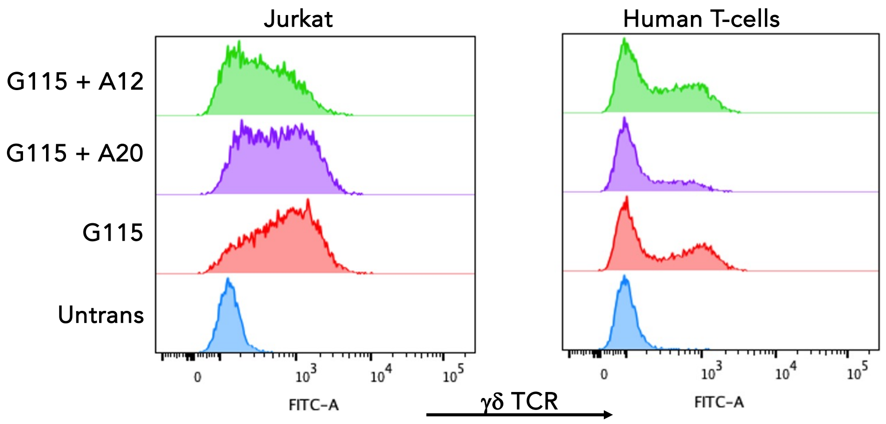

B

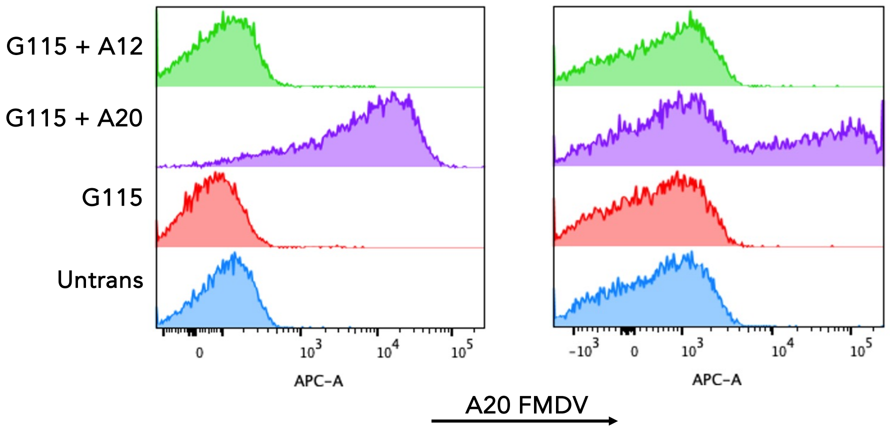

Supplementary Figure S2. Detection of expression of G115-derived TCRs using an A20 FMDV-specific antibody. Representative flow cytometry plots that demonstrate cell surface expression of G115 TCRs containing the indicated peptide insert in Jurkat cells and human T-cells following transduction with the indicated construct, making comparison with untrans(duced) cells. Cells were incubated with a pan γδ TCR antibody (**A**) or an anti-A20 FMDV monoclonal antibody (**B**). Data are representative of 3 independent replicates.

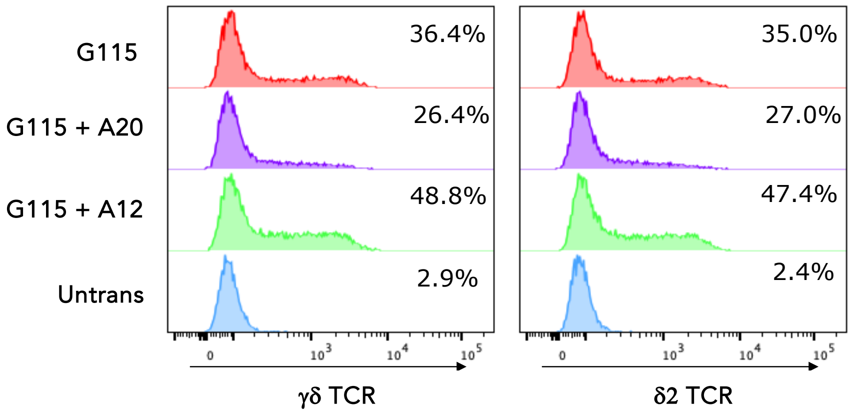

Supplementary Figure S3. Detection of expression of G115-derived TCRs using a pan γδ TCR or δ2 chain-specific antibody. Representative flow cytometry plots that demonstrate cell surface expression of G115 TCRs containing the indicated peptide insert human T-cells, making comparison with untrans(duced) cells. Data are representative of 3 independent replicates.

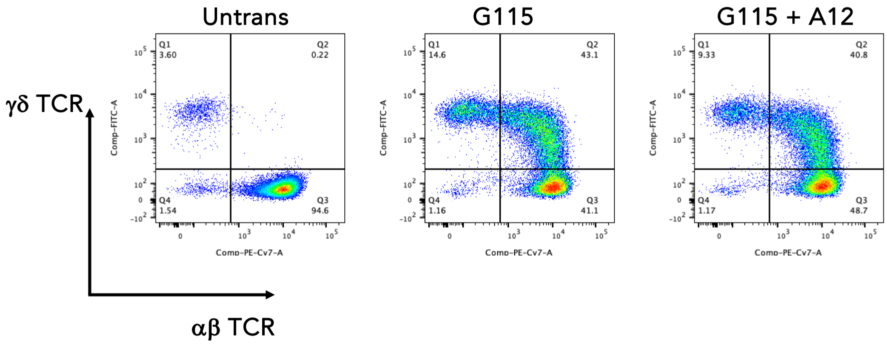

Supplementary Figure S4. Expression of the endogenous αβ TCR in in human T-cells following transduction with the G115 or G115 + A12 γδ TCRs, making comparison with untrans(duced) T-cells. Representative flow cytometry plots that demonstrate cell surface γδ and αβ TCR expression in human T-cells following transduction with the indicated contruct. Data are representative of 3 independent replicates.

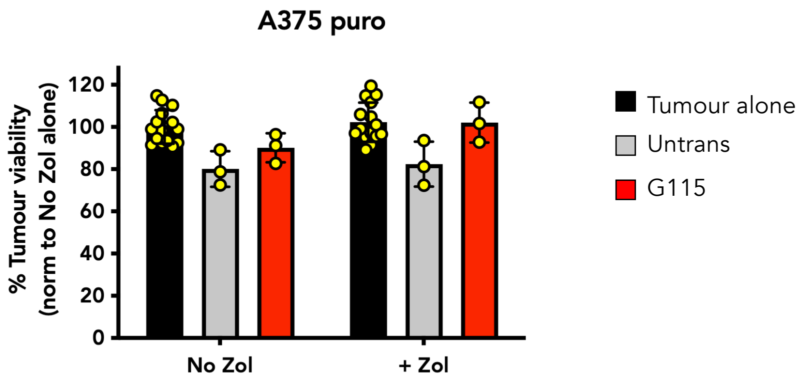

Supplementary Figure S5. Cytotoxicity assay. A375 puro cells were co-cultivated with G115 transduced or untrans(duced) T-cells at an effector to target ratio of 1:1 for 72 hours. Residual tumour viability was then determined by MTT assay, making comparison with tumour alone (mean $\pm$SEM of 3-15 replicates).

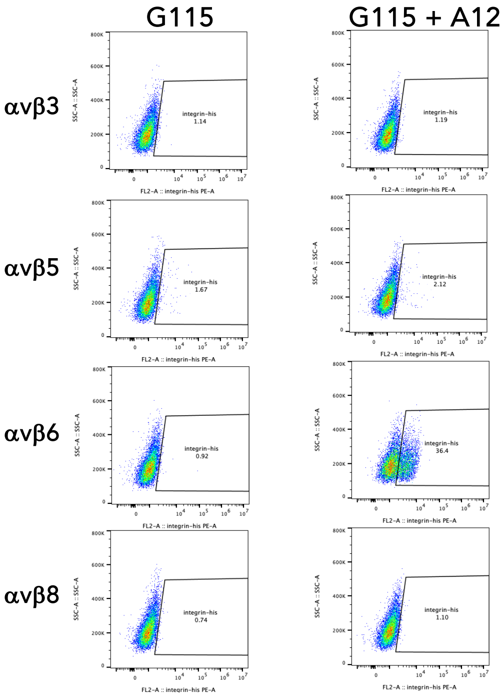

Supplementary Figure S6. Integrin binding by human T-cells engineered to express G115-derived TCRs. Representative flow cytometry plots that demonstrate binding of the indicated His-tagged integrins to G115 or G115 + A12-engineered human T-cells. Gates were set using untransduced T-cells. Data are representative of 3 independent replicates.

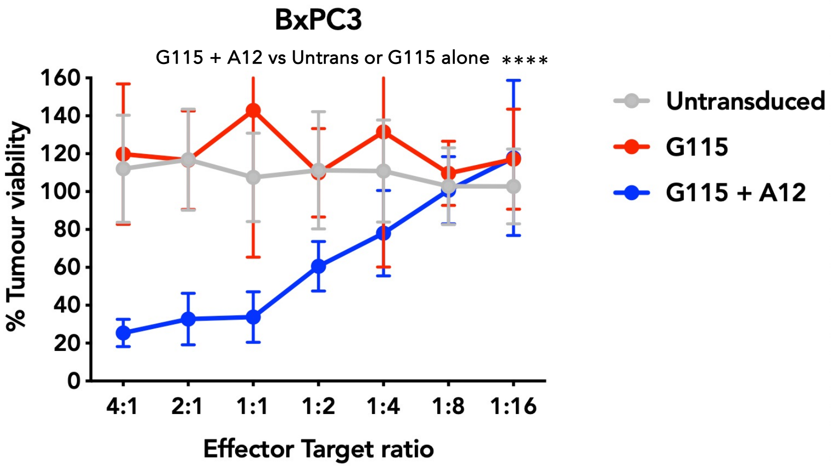

Supplementary Figure S7. Cytotoxicity assay. BxPC3 cells were co-cultivated with G115, G115 + A12 transduced or untrans(duced) T-cells at the indicated effector to target ratio for 72 hours. Residual tumour viability was then determined by MTT assay, making comparison with tumour alone (mean ± SEM of 6 replicates from 2 independent donors).

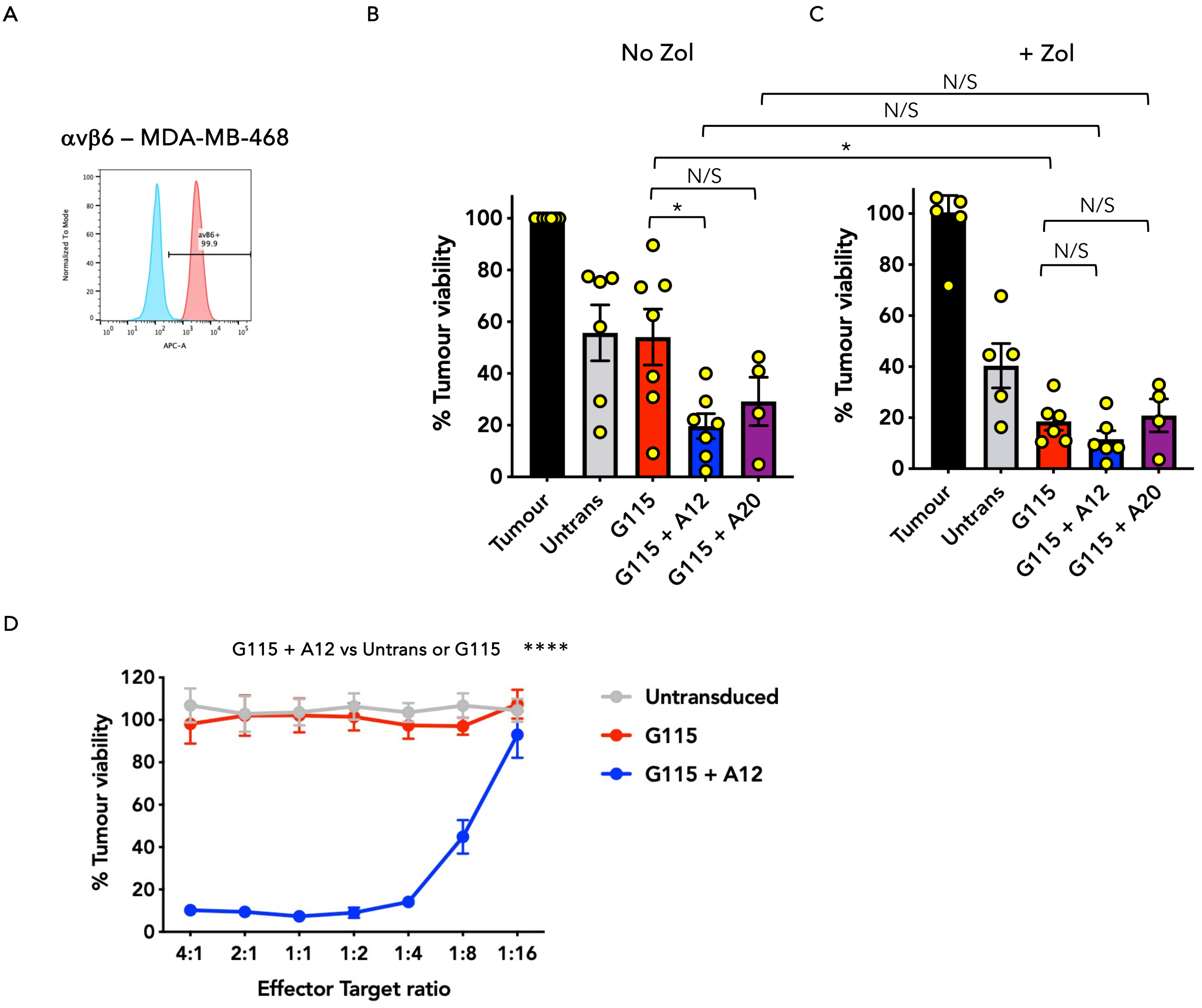

Supplementary Figure S8. Evaluation of anti-tumour activity of chimeric G115 γδ TCRs against MDA-MB-468 triple negative breast cancer cells. (**A**) Analysis of αvβ6 integrin expression on MDA-MB-468 cells. Red – integrin; blue – isotype control. Data are representative of 3 independent replicates. Co-cultures were performed between MDA-MB-468 tumour cells (No Zol; **B**) or Zol-sensitised MDA-MB-468 tumour cells (+ Zol; **C**) and untrans(duced) or transduced T-cell populations at an effector : target ratio of 1:1 for 72 hours. Tumour cell viability was determined using an MTT assay (mean +_SEM of indicated replicates). Statistical analysis was performed using one-way ANOVA; **p*<0.05, N/S – not significant. (**D**) MDA-MB-468 cells were co-cultivated with G115, G115 + A12 transduced or untrans(duced) T-cells at the indicated effector to target ratio for 24 hours. Residual tumour viability was then determined by MTT assay, making comparison with tumour alone (mean +_ SEM of 6 replicates from 3 independent donors).

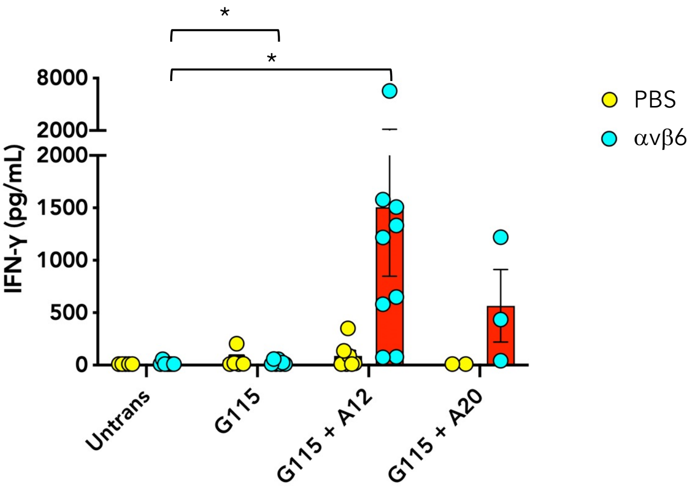

Supplementary Figure S9. Release of IFN-γ by chimeric G115 γδ TCRs upon stimulation with immobilised αvβ6 integrin. The indicated engineered T-cell populations and untransduced (untrans) T-cells were stimulated on immobilised αvβ6 integrin, making comparison with PBS. Supernatants were analysed for IFN-γ content after 72 hours (mean ± SEM). Statistical analysis was performed using two-way ANOVA; *$p<0.05$.
